# Postbiotics in Dairy: A Comprehensive Review of Applications and Health Impacts

**DOI:** 10.1007/s13668-026-00767-z

**Published:** 2026-04-30

**Authors:** Yasemin Kaya, Haktan Aktaş, Hacer Meral-Aktaş, Bayram Ürkek, Zeynep Gürbüz-Kaçan, Esengül Çiftçi, Tuba Erkaya-Kotan, Mustafa Şengül, Bülent Çetin

**Affiliations:** 1 Faculty of Agriculture, Department of Food Engineering, Ataturk University, Erzurum, 25240 Turkey; 2 Gumushane University, Siran Mustafa Beyaz Vocational School, Siran, Gumushane, 25700 Turkey

**Keywords:** Postbiotic, Health, Dairy products, Microorganisms, Metabolites

## Abstract

**Purpose of Review:**

This review aims to provide a comprehensive overview of postbiotics—defined as non-viable microbial cells, cell components, and microbial metabolites—and their emerging role in dairy products. It focuses on their functional, technological, and health-promoting properties in various dairy matrices, including yoghurt, cheese, kefir, fermented milk, and ice cream. Additionally, the review highlights the potential of postbiotics as stable and safe alternatives to probiotics in the development of functional dairy foods.

**Recent Findings:**

Recent evidence demonstrates that postbiotics such as exopolysaccharides (EPS), bacteriocins, short-chain fatty acids (SCFAs), bioactive peptides, and organic acids significantly enhance both the nutritional value and technological quality of dairy products. These compounds have been shown to improve texture, sensory attributes, shelf life, and microbial safety while exerting biological activities, including antimicrobial, antioxidant, immunomodulatory, and gut health–promoting effects. Compared with probiotics, postbiotics offer greater stability during processing and storage as well as reduced safety concerns, particularly for vulnerable populations.

**Summary:**

Postbiotics represent a promising strategy for advancing functional and sustainable dairy products by combining health benefits with technological improvements. Their applications in functional food formulation, biopreservation, and bioactive compound delivery are rapidly expanding. However, challenges remain regarding standardised production processes, accurate quantification of bioactive components, and clinical validation of health claims. Future research should focus on optimising postbiotic production and strengthening evidence for their physiological effects to support their broader incorporation into next-generation dairy products.

## Introduction

**Fig. 1:**
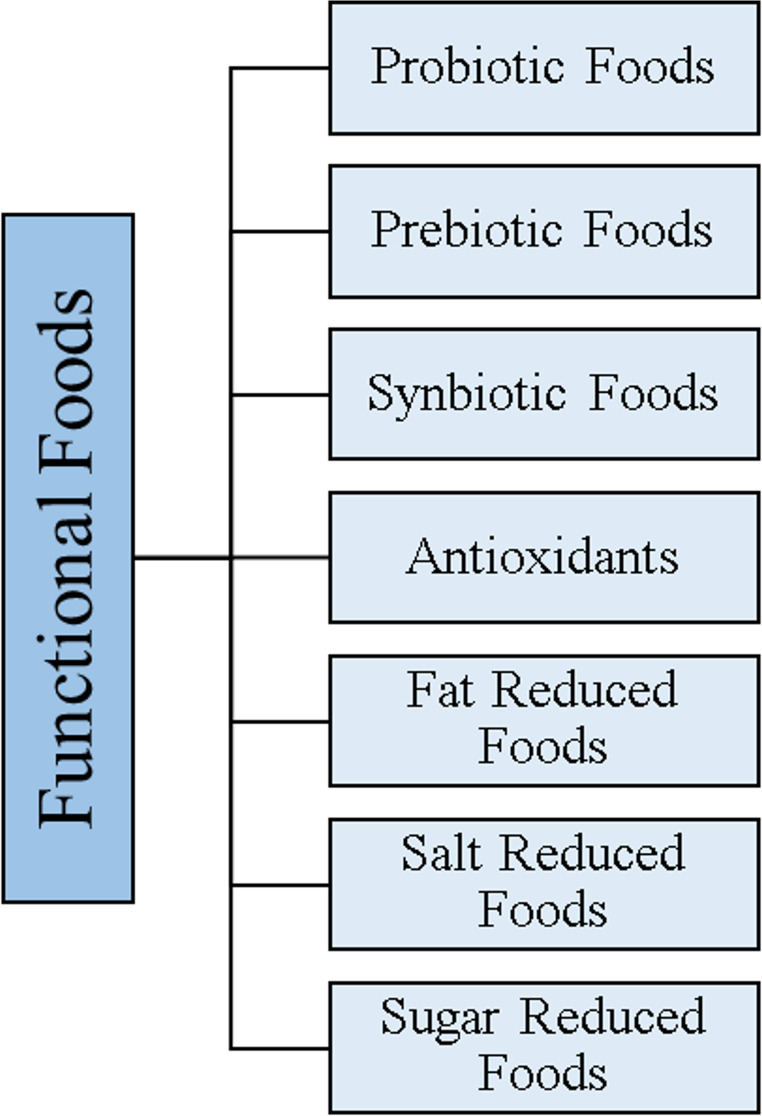
Classification of functional foods [8]. Schematic overview of major categories of functional foods, including probiotic, prebiotic, and synbiotic foods, as well as products with added health-promoting components (e.g., antioxidants) and modified compositions such as fat-, salt-, and sugar-reduced foods

The relationship between nutrition and healthy life has been known for many years [1]. Researchers reported that adequate and balanced nutrition can prevent many diseases, such as cancer, cardiovascular diseases [2], diabetes [3], immune system diseases [4], obesity [5], and Parkinson’s [6]. In accordance with this information, consumers’ perspectives on food, nutrition, and healthy life have changed at the beginning of the 21st century. Consumers expect food not only to relieve hunger but also to prevent diseases and even increase human health. These expectations have created functional food in the food industry [7]. Functional foods are defined as foods that can have positive effects on human health, in addition to their basic nutritional functions [8, 9]. Today, many foods are included in functional foods (Fig. 1).

Fermented products such as yoghurt, kefir, cheese, fermented milk, pickles, fermented cereals, and sausage are the most important group in functional foods due to their health effects and excess consumption. Fermentation is the biochemical change in raw material as a result of biological activity by microorganisms such as lactic acid bacteria (LAB), yeast and mould; moreover, fermentation, whose history dates back to 6000 BC, is one of the best preservation methods [10, 11]. Among the fermented foods, fermented milk products compose an important part of the human diet [12]. Many studies have shown that these products can prevent inflammatory disorders, cardiovascular and gastrointestinal diseases, dysbiosis, diabetes, osteoporosis, and *Helicobacter pylori* infections. These positive effects on health are mostly due to the microorganisms such as LAB, yeast, and mould and their metabolites [13]. Some of these microorganisms, defined as probiotics, can survive in the human gastrointestinal tract and provide beneficial effects on health, such as anti-*Helicobacter pylori* benefits, prevention of inflammatory diseases, bowel syndromes, cancer, and constipation, and modulation of the immune system [14]. On the other hand, metabolites by these microorganisms or their dead cell materials can also have positive effects on human health (postbiotics) [15].

According to the International Scientific Association of Probiotics and Prebiotics (ISAPP), postbiotics are defined as “preparations of inanimate microorganisms and/or their components that confer a health benefit on the host”. Postbiotics include non-viable microorganism cells, microbial metabolites, and components released from the lysed cell. On the other hand, vaccines and purified metabolites are not included in postbiotics [15].

Postbiotics do not have to be produced only by probiotics; moreover, they have significant advantages over probiotics. Probiotics must maintain their viability and must be transported and stored in the cold chain. On the contrary, postbiotics are more stable than probiotics and easier to transport and store [16, 17]. Consumption of probiotics for immunocompromised individuals, the elderly, and newborns may cause septicaemia by passing from the intestine to the blood [18]. In addition, probiotics may play a role in the transfer of virulence and pathogenicity genes in the gastrointestinal tract [19]. Furthermore, probiotics take time to colonize the colon and synthesize their metabolites in order to exert their health benefits [20]. Some researchers reported that microbial viability is not required for the effects of microorganisms on health [21]. Postbiotics do not need to be taken in very high numbers to show their effects, they are easy to prepare and have a targeted effect [22, 23]. Another advantage of postbiotics is that they can be produced in large quantities by cloning technologies [24].

**Fig. 2:**
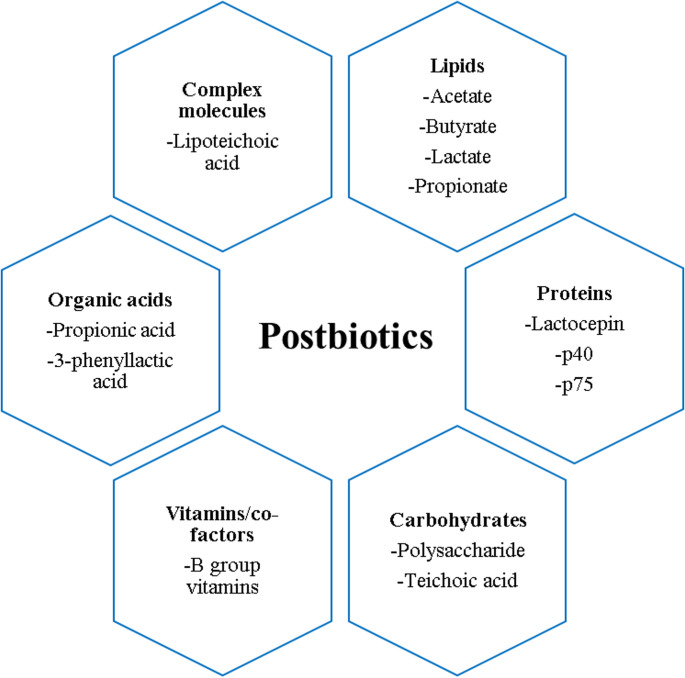
Classification of postbiotics. Overview of major postbiotic components derived from microbial metabolism, including organic acids, lipids, proteins, carbohydrates, vitamins/cofactors, and complex molecules, with representative compounds presented for each category

Postbiotics are classified according to their elemental structure, such as proteins, carbohydrates, lipids, vitamins/cofactors, organic acids, and complex molecules (Fig. 2) [25, 26]. Postbiotics can also be classified by their molecular weight such as low (H_2_O_2_, CO_2_, reuterin, organic acids) and high molecular weight compounds (bacteriocins, bacteriocin like substances) [23, 27]. Another classification is based on their physiological effects. Accordingly, they can be classified as antioxidant, antitumour, antimicrobial, immunomodulatory, and hypocholesterolaemic agents [28].

It is known that postbiotics have beneficial effects on human health. Moreover, the beneficial effects on the host (such as humans, experimental animals, and farm animals) must be proven [15]. For this reason, many studies have been carried out on this subject. As a result, it has been determined that postbiotics have beneficial effects on human health such as anticancer [26], immunomodulation [23, 25, 29–32], anti-inflammatory [25, 32], antimicrobial [23, 25], antioxidant [23, 25, 32], antihypertensive, hypocholesterolaemic [25], wound healing [33], prevention of colon diseases [34], coronary artery diseases and osteoporosis [35], and relieving symptoms of some diseases and viral infections [23].

Additionally, postbiotic usage in food production can increase shelf life and nutritional value. Researchers have stated that postbiotics can be used in the preparation of functional foods [36], the packaging of foods [37], as biopreservatives [38, 39], for removing biofilms by pathogens [40, 41], improving sensory properties [42], improving the rheological properties [43], and removing toxic components [44]. This review aims to provide a comprehensive overview of the potential health-related effects of postbiotics, based on evidence from in vitro, in vivo, and available clinical studies, with a particular focus on their applications in dairy products. It should be noted that most of the current evidence is derived from in vitro and animal studies, and clinical validation in humans remains limited.

## Yoghurt as a Potential Postbiotic Source

Yoghurt is one of the most widely consumed fermented dairy products, obtained by the fermentation of milk using *Lactobacillus delbrueckii* subsp. *bulgaricus* and *Streptococcus thermophilus* [45]. Due to their well-documented health-promoting effects, probiotics are widely incorporated into yoghurt production. Indeed, yoghurt serves as a suitable environment for the growth of probiotics. These microorganisms have been reported to contribute to the management and prevention of various conditions, including colitis, diarrhoea, lactose intolerance, irritable bowel syndrome, insulin resistance, hypertension, hypercholesterolaemia, certain cancers, allergies, and obesity [46–51]. These health-promoting effects have been reported as highly strain-specific; however, recent studies have shown that they also depend on consumption methods, frequency and dose [52]. The traditional definition of probiotics implies that bacteria must be alive or viable to exert their health-promoting effects. However, current data demonstrate that microbial viability is not required to obtain such effects [53]. Some researchers have suggested that bacterial metabolites could potentially induce the desired effects and offer a safer alternative in the treatment of inflammatory diseases [54]. As yoghurt starter cultures can survive in the human gastrointestinal tract, they are primarily considered probiotic strains [55, 56]. However, all strains of these bacteria vary in different regions of the world; therefore, their probiotic potential is still controversial [57].

On the other hand, recent studies have explored the potential for *S. thermophilus* and *L. bulgaricus* to produce postbiotics (Table 1). During the fermentation process, lactic acid and many metabolites such as bioactive compounds, exopolysaccharides (EPS), short-chain fatty acids, enzymes, peptides, organic acids and vitamins are produced by these starter bacteria as postbiotics. Numerous studies have indicated the potential health effects of various postbiotics produced by many strains of bacteria including yoghurt starters. A summary of several studies investigating postbiotics produced by yoghurt bacteria with their health benefits is depicted in Table 1.

**Table 1:** Postbiotics produced by yoghurt-associated bacteria and their functional properties

Yoghurt bacteria	Postbiotics	Health benefits	References
*S. thermophilus* ST538	EPS	Modulation of antiviral immune response	[58]
*L. delbrueckii* subsp. *bulgaricus* OLL1073R-1	EPS	Immunomodularity and immunostimulatory effect	[59–61]
*L. delbrueckii* subsp. *bulgaricus*, *S. thermophilus*	Bile salt hydrolase, antimicrobial agents, EPS	Immunomodulatory effect	[62]
*S. thermophilus* CRL 1190	EPS	Therapy for chronic gastritis	[63]
*L. delbrueckii* subsp. *bulgaricus* ATCC 11842, *S. thermophilus* ATCC 19258	Intracellular component	Antioxidant activity	[64]
*L. delbrueckii* subsp. *bulgaricus B3*	EPS	Cholesterol reduction	[65]
*L. delbrueckii* subsp. *bulgaricus* BGVLJ1-21, *S. thermophilus* BGKMJ1-36	EPS, antimicrobial peptides, lactic acid	Improving gut epithelial barrier	[66]

*EPS:* exopolysaccharides, *L. delbrueckii* subsp. *bulgaricus*: *Lactobacillus delbrueckii* subsp. *bulgaricus*, *S. thermophilus*: *Streptococcus thermophilus*

As seen in Table 1, postbiotics produced by yoghurt bacteria may aid in managing conditions related to immune function, gut health, antimicrobial and antioxidant activity, and cholesterol metabolism. Several studies have reported that consumption of fermented foods containing LAB, such as yoghurt, may contribute to overall health and well-being [12, 13]. The production of postbiotics by these microorganisms is considered to play an important role in these observed health-related effects [31, 52].

### Exopolysaccharides (EPSs)

EPSs are complex carbohydrates composed of different monosaccharide units [67]. *L. delbrueckii* subsp. *bulgaricus* and *S. thermophilus* have the ability to produce EPS during yoghurt fermentation. In recent years, there has been increased interest in using the EPSs secreted by these bacteria to improve the texture, viscosity, and mouthfeel of yoghurt and other fermented dairy products [67, 68]. Moreover, some studies reported that EPSs have functional health attributes beyond their rheological and organoleptic effects [69]. EPSs are derived in different ways in fermented foods. Wzx/Wzy-dependent pathways, which involve the assembly and transport of polysaccharide units across the cell membrane via specific flippase (Wzx) and polymerase (Wzy) proteins, are used by *L. delbrueckii* subsp. *bulgaricus* and *S. thermophilus* for homoexpolysaccharide formation, while the extracellular synthetic pathway is used for heteropolysaccharide production [70]. To date, a number of EPS-producing *S. thermophilus* and *L. delbrueckii* subsp. *bulgaricus* strains have been isolated and identified [71–73]. Li and Shah [73] investigated the antimicrobial and antioxidant activities of sulphated EPSs from *S. thermophilus* ASCC 1275 and reported enhanced bioactivity after sulphation. However, these findings are based on in vitro assays, and their relevance under physiological conditions remains unclear. Furthermore, differences in EPS structure and assay conditions across studies make direct comparison of antioxidant capacity challenging. Similarly, Zhang et al. [74] indicated that sulfated EPS from *S. thermophilus* GST-6 has stronger inhibitory efficacy than non-sulfated EPS against *Escherichia coli*, *Salmonella* Typhimurium, and *Staphylococcus aureus.* Nevertheless, variations in experimental design and microbial targets across studies may influence the reported antimicrobial potency, limiting the ability to directly compare these findings with other reports. As mentioned in Table 1, various EPSs produced by different strains of *L. delbrueckii* subsp. *bulgaricus* and *S. thermophilus* have been proposed to have immunomodulatory effects. In this context, Kanmani et al. [71] found that EPS from *L. delbrueckii* OLL1073R-1 helped to improve intestinal innate response and enhance antiviral immunity specifically against rotaviruses.

Yoghurt fermented with probiotic bacteria or probiotic strains of yoghurt starter cultures has a great potential for the production of postbiotics. Makino et al. [59] demonstrated that daily intake of 90 g yoghurt fermented with *L. bulgaricus* OLL1073R-1 over 8–12 weeks in elderly populations (*n* = 57 and 85) significantly reduced the incidence of the common cold, with an approximately 2.6-fold lower risk compared to the control group (OR = 0.39; *P* = 0.019). Similarly, Nagai et al. [75] investigated the anti-influenza virus effects of yoghurt and revealed that oral administration of EPS (postbiotic) and yoghurt fermented with *L. bulgaricus* OLL1073R-1 and *S. thermophilus* OLS3059 had anti-influenza virus effects in mice via immunopotentiating action, thus, the yoghurt might protect against influenza virus infection in vivo.

### Bioactive Compounds

Studies conducted in vitro and in vivo have shown that postbiotics formed in fermented dairy products, such as yoghurt, exhibit bioactive properties. During fermentation, many different strains of LAB can produce bioactive substances in yoghurt like bioactive peptides, glutathione, β-glucan, and γ-aminobutyric acid (GABA), which have medicinal and health-beneficial effects [76]. For instance, a study investigating the optimisation of the production of high amounts of the free form of protease of *S. thermophilus* 4F44 has described its activity on sodium caseinate to produce potential bioactive peptides [77]. The researchers have characterised twenty-two bioactive peptides, including ACE-inhibitory, antioxidant, immunomodulating, or antibacterial peptides and they suggested that the addition of such peptides could improve the health benefits of dairy products. Although numerous studies report ACE-inhibitory and antioxidant activities of postbiotics derived from different microbial strains, the magnitude of these effects varies considerably depending on several factors, including strain specificity, fermentation conditions, substrate composition, and processing parameters. In general, bioactivity is assessed using different analytical approaches such as IC_50_ values for ACE inhibition, and DPPH, ABTS, or FRAP assays for antioxidant capacity. However, direct comparison between studies remains challenging due to the lack of methodological standardisation, differences in experimental models (in vitro vs. in vivo), and variations in reporting units. Some strains, particularly *Lactobacillus helveticus*, *Lacticaseibacillus casei*, and *Streptococcus thermophilus*, have been reported to produce postbiotics with relatively higher ACE-inhibitory and antioxidant activities. Nevertheless, it is difficult to establish a clear prioritisation among strains, as results are highly context-dependent and influenced by the food matrix and fermentation conditions. Therefore, caution should be exercised when comparing outcomes across studies, and further research is needed to standardize evaluation methods and enable more reliable comparisons of postbiotic potency. In another study by Linares et al. [78], *S. thermophilus* APC151 was used as in yoghurt fermentation as a potential GABA-producing strain, isolated from the digestive tract of a fish, and the yoghurts were compared with control yoghurts produced by commercial starters. The researchers obtained yoghurt that was naturally enriched with GABA (2 mg/ml), while no GABA production was detected in the control yoghurt. Therefore, they determined that the use of the aforementioned strain was a natural and effective way to enhance yoghurt with GABA [78]. However, GABA production levels may vary significantly depending on strain selection and fermentation conditions, and the bioavailability of GABA after digestion remains insufficiently explored. Similarly, Atasoy and Şengül [79] demonstrated that the addition of postbiotics derived from *Lacticaseibacillus casei* ATCC 393 to yoghurt increased its antioxidant activity and ACE-inhibitory capacity.

Supplementation with probiotics and prebiotics leads to a significant increase in the production of bioactive compounds in yoghurts. Probiotics have traditionally been added to yoghurt, and currently, there are over 70 products worldwide that contain lactobacilli or bifidobacteria, including sour cream, buttermilk, frozen desserts, and probiotic drinks [80]. A recent study showed that a functional synbiotic yoghurt enriched with *Levilactobacillus brevis* KU200019 and fructooligosaccharides (FOS) had high antioxidant, ACE-inhibitory, reactive oxygen species scavenging, and immunomodulatory activities owing to the postbiotics generated by this bacterium. The study demonstrated enhanced antioxidant and ACE-inhibitory activities in synbiotic yoghurt. Nevertheless, these effects may not be solely attributed to postbiotics, as the interaction between probiotics and prebiotics (synbiotic effect) could also contribute to the observed bioactivity, complicating the interpretation of results [81]. A significant increase in ACE-inhibitory and DPPH-radical-scavenging activities has been reported in probiotic set yoghurt produced by the use of ABT-2 culture (which includes *S. thermophilus* ST-20Y, *Lactobacillus acidophilus* LA-5, and *Bifidobacterium animalis* spp. *lactis* BB-12) [82]. This increase was observed with the addition of orange fibre levels in the yoghurt. In a similar study, Darwish et al. [36] produced a functional yoghurt enriched with Cape gooseberry using *E. coli* Nissle 1917, with the combination of yoghurt bacteria and investigated the antimicrobial, antitumor, and antioxidant activities of postbiotics derived from *E. coli* Nissle 1917. The authors noted that the antimicrobial, antitumour, antioxidant, total phenolic content, and survival of *E. coli* Nissle 1917 significantly increased during yoghurt storage. They proposed that the use of polyphenol-rich products in the production of dairy products could improve these properties.

### Bacteriocins

Bacteriocins are extracellular antimicrobial peptides that are generated as primary metabolites in ribosomes by phylogenetically diverse bacteria and archaea and are thought to be important sources of gut microbiota biodiversity [83]. Probiotics can produce bacteriocins that can inhibit the growth of closely related pathogenic species and other microorganisms causing damage. Due to their broad applications in food processing and fermentation, they are popular as natural bio-preservatives [84]. Previous studies have demonstrated the antibacterial activity of bacteriocins produced by *L. delbrueckii* subsp. *bulgaricus* strains isolated from yoghurt against various foodborne pathogens and spoilage microorganisms, particularly *Vibrio cholerae* and *E. coli* [85]. According to the study, the highest level of bacteriocin production was achieved after 48 h of incubation. In a different research, a synbiotic yoghurt was produced by adding inulin and FOS to a commercial yoghurt containing *L. acidophilus* and its bacteriocins [86]. The study results showed higher quality attributes of the yoghurts such as coagulation time, shelf life extension, and microbiological and sensory characteristics when compared to plain yoghurt. Also, Jiang et al. [87] identified a novel bacteriocin, LSX01, produced by *Lacticaseibacillus paracasei* LS-6 isolated from a traditional fermented yoghurt in Yunnan, China. In another study, researchers investigated the inhibition effect of the usage of bacteriocin-producing *S. thermophilus* B and *L. delbrueckii* subsp. *bulgaricus* CY (isolated from commercial yoghurt) on *Listeria monocytogenes* and *S. aureus* during fermentation and storage of yoghurt. The in vitro study revealed that *S. aureus* is less sensitive to the bacteriocin by *S. thermophilus B* compared to *L. monocytogenes* [88].

### Short Chain Fatty Acids, Organic Acids, Vitamins and Volatile Compounds

SCFAs are microbial metabolites that are naturally formed in the human colon through bacterial fermentation of nondigestible carbohydrates via multiple mechanisms [89]. Probiotic microorganisms produce SCFAs in the human colon by fermenting indigestible carbohydrates (sucrose, propionate, butyrate). Chang et al. [89] determined the SCFA levels in yoghurt using different probiotic combinations with yoghurt starter bacteria. They found that yoghurt fermented with *Bifidobacterium bifidum*, *L. acidophilus*, or *Lactobacillus gasseri* contained significant levels of SCFA, especially acetate and proposed that an acetate-rich yoghurt diet increases the protective function of the intestinal epithelium.

In recent years, *Saccharomyces boulardi* as a probiotic yeast has gained popularity because of its unique physiological traits, as well as genomic and phenotypic characteristics, health advantages, and mechanisms of action [90]. Recent reviews have highlighted the technological potential of *S. boulardii* in some food matrices compared to conventional probiotic bacteria [91, 92]. These food matrices include dairy foods such as yoghurt, whey and ice cream. Co-culturing of *S. boulardi* with LAB and producing synbiotics with prebiotics may increase the quantity of metabolites in postbiotics in dairy products. Mehaya et al. [93] developed soy milk yoghurt with the co-culturing of *S. boulardii* CNCMI-745 and *Lactiplantibacillus plantarum* KU985432 and showed that *Saccharomyces*-yoghurt was a potential source of bioactive compounds such as phenolic acids, fatty acids, and B-vitamins such as thiamine, riboflavin, and pyridoxine, as well as improving the bioavailability of key minerals. Similarly, Sarwar et al. [94] produced a synbiotic yoghurt with *S. boulardii* and inulin. The combination of yeast and inulin increased antioxidant and favourable volatile compounds compared with the probiotic and control plain yoghurt.

Finally, while *S. boulardii* or other probiotic microorganism postbiotics in yoghurt have shown potential health benefits in laboratory and animal studies, it’s important to note that these studies don’t take into account how these postbiotics would function in a human host. This is because postbiotic-containing foods that are rich in anti-pathogenic enzymes, proteins, and peptides may lose their effectiveness after being digested by the host’s enzymes. Therefore, clinical studies are necessary to determine whether these postbiotic preparations can actually lead to measurable health improvements, especially if a health claim is being made.

## Cheese as a Potential Postbiotic Source

**Fig. 3:**
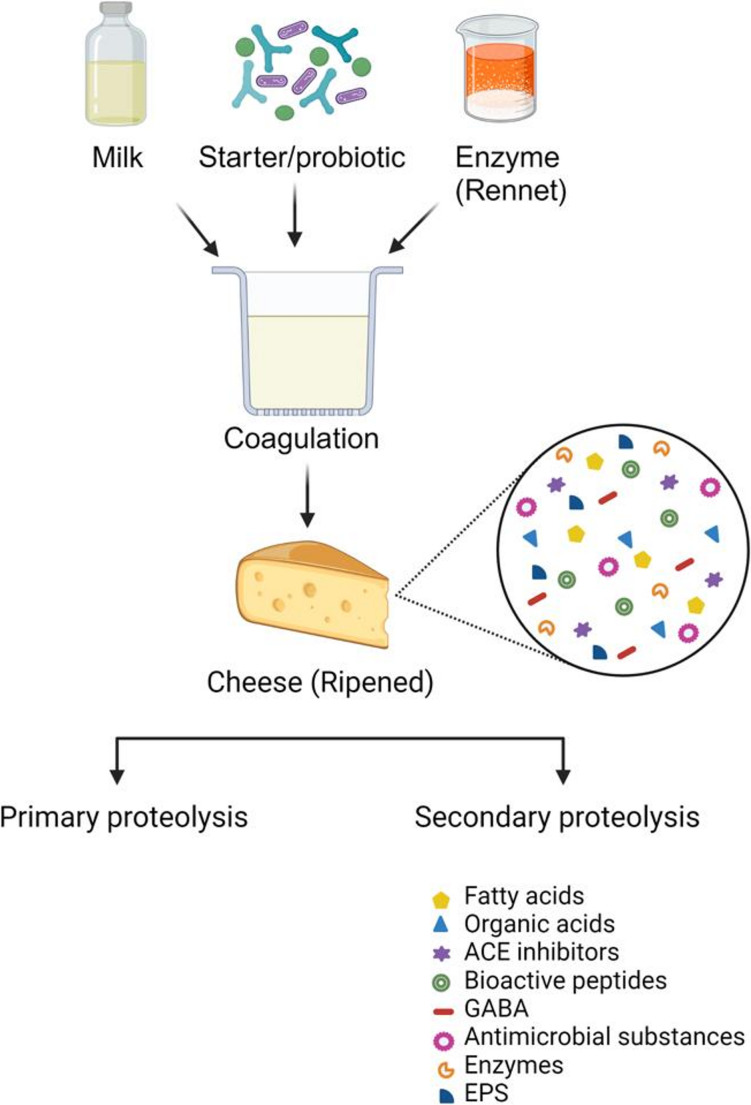
Postbiotics in cheese. Schematic representation of cheese production and ripening, illustrating the role of starter/probiotic microorganisms and enzymes in coagulation, followed by primary and secondary proteolysis leading to the formation of postbiotic compounds such as bioactive peptides, organic acids, fatty acids, GABA, antimicrobial substances, enzymes, and EPS

Cheese is a nutritious and functional dairy product that can be consumed either fresh or ripened and is produced by coagulation of the milk protein [95, 96]. The important product is source of nutrients such as proteins, SCFAs, carotenoids, prebiotics, probiotics, vitamins, and minerals [97–99]. Moreover, some studies reported that cheese matrix was effective for protection of probiotics during their passage through the gastrointestinal tract [100–103]. For this reason, cheese is also important in terms of postbiotics produced by probiotics or other microorganisms (Fig. 3). Some studies on probiotic and postbiotic cheese production are summarised in Table 2.

**Table 2:** Summary of studies on probiotic and postbiotic applications in cheese production and their effects on product properties

Cheese types	Probiotic strains	Effect of probiotics on cheese	References
Wagashi cheese	*Lacticaseibacillus casei* 39, *Lacticaseibacillus rhamnosus*,* L. plantarum*,* Enterococcus faecium* K65E2	Antioxidant activity, organic acids	[96]
Dutch Edam cheese	*L. casei* LAFTI-L26	High proteolytic and lipolytic activity, well textural property, high sensory evaluation	[104]
Cheddar cheese	*L. casei* 1.0319, *L. rhamnosus* 1.0911, *Lactobacillus helveticus* 1.0612, *Lactococcus lactis* subsp. *cremoris*,* Lactococcus lactis* subsp. *lactis*	High ACE-I activity, high proteolytic activity, lower the molecular mass	[105]
Cheddar cheese	*L. helveticus* 1.0612, *L. rhamnosus* 1.0911, *L. casei* 1.0319	High ACE-I activity	[106]
Cheddar cheese	*L. rhamnosus* DPC7102, *L. paracasei* DPC7150	Long shelf life	[107]
Prato cheese	*L. lactis* ssp. *Lactis*,* L. lactis* ssp. *cremoris*,* L. casei-01*	High content of amino acids (leucine, isoleucine, and valine), fatty acids, and aromatic compounds (tyrosine, phenylalanine, histidine, benzoic acid, and formic acid)	[95]
Ricotta cheese	*L. paracasei* BGP1	Long shelf life, more stable physicochemical properties	[108]
Fresh cheese	*L. plantarum* 299v, *Bifidobacterium animalis* Bo	Reduced hepatocyte lipidic accumulation, increased adipolysis, anti-inflammatory effect on macrophages	[109]
Chami (traditional soft cheese)	*Pediococcus pentosaceus*	High α-glucosidase, α-amylase and DPP-IV inhibitory activity	[99]
Semi-hard Spanish sheep’s milk cheeses	*Lactococcus lactis* INIA 650 pT1.aFP, *L. paracasei* INIA P272, *Bifidobacterium breve* INIA P734	Nisin production, more stable physicochemical and sensory properties	[110]

### Bioactive Peptides

Cheese is a good source not only of bioavailable proteins, but also of various bioactive peptides produced by enzymatic proteolysis or bacterial fermentation [111–113]. Bioactive peptides are important postbiotics found in a variety of foods consumed daily and have no known side effects. Most bioactive peptides are found in proteins but are inactive and activated by bacterial activity during food processing or digestion [114].

Bioactive peptides in cheese are formed as a result of secondary proteolysis. Firstly, the breakdown of casein into large peptides by rennet and natural milk proteinases is called primary proteolysis, while hydrolysis of the large peptides into peptides and amino acids by microbial proteinases and peptidases is called secondary proteolysis [115]. Since there are many factors for formation of bioactive peptides, starter culture, cheese type, and ripening conditions play an important role in the formation and concentration of bioactive peptides [116, 117]. In the study by Amiri et al. [104], the effects of co-culture of *L. casei* LAFTI-L26 on traditional Edam cheese were investigated, and the results showed that the co-cultured cheese contained more low molecular weight peptides than control samples. As a result, it was concluded that bacterial enzymes could cause advanced secondary proteolysis, which affects flavour, aroma, and quality of the cheese.

Öztürk and Akın [118] investigated the peptide profile of 180-day-ripened Tulum cheeses, which are traditionally made in Turkey’s Central Taurus region. After the study, 203 peptides were found to be bioactive; these included peptides that were antihypertensive, antibacterial, antioxidant, dipeptidyl peptidase-4 inhibitory, metal chelating, skin regenerating, enhancing the secretion of glucagon-like peptide-1, opioid, cathepsin B inhibitory, prolyl endopeptidase inhibitory, immunomodulatory, improving brain function, antiamnesic, antihypercholesterolaemic, anti-inflammatory, and carcinogenic. However, the functional relevance of many of these peptides remains unclear, as most studies focus on identification rather than quantification or in vivo validation of their biological effects.

### Angiotensin-converting Enzyme Inhibitors and Glucagon-like Peptide-1

ACE inhibitory peptides play an important role in blood pressure regulation [119, 120]. In addition to their antihypertensive effects, some bioactive peptides derived from dairy products also exhibit antioxidant activity, which may further contribute to cardiovascular health by reducing oxidative stress and preventing conditions such as coronary heart disease, atherosclerosis, and arthritis [121]. In the study by Baptista et al. [122], the bioaccessibility of bioactive peptides in Prato cheese with *L. helveticus* LH-BO2 was investigated. As a result of the study, αs1-CN (f18-23), αs1-CN (f24-32), αs1-CN (f1-9), β-CN (f199-209), β-CN (f195-206), β-CN (f194-209), and β-CN (f193-209) peptides (ACE inhibitors), dipeptidyl peptidase IV (DPP-IV) inhibitor, antihypertensive, glucagon-like peptide-1 (GLP-1), and components exhibiting immunomodulatory, antithrombin and antimicrobial activities were detected in the cheese and digestive system. Thus, it was stated that cheese could have bioactive components, but the mechanism of action of the bioactive peptides is not yet fully understood and in vivo tests are still needed. Additionally, the bioaccessibility and stability of these peptides during digestion may significantly affect their physiological efficacy, highlighting the need for more in vivo studies. In another study on ACE-I peptides of cheddar cheese with probiotic microorganisms, it was found that ACE-I activity of samples *L. helveticus* was higher that of other cheeses [105]. Mushtaq et al. [123] exposed *Kradi* cheese to a simulated gastrointestinal digestion environment and found that water-soluble peptide extract obtained after the application had higher inhibition of 2,2-azinobis(3-etilbenzothiazollin-6-sulfonik asit) (ABTS) radical, lipid peroxidation, α-glucosidase and α-amylase. Moreover, anti-cancer (from 13.44 to 69.39%) and anti-hypertensive activity (from 19.75 to 71.23%) of the water-soluble peptide extract increased significantly after digestion in simulated gastrointestinal digestion environment.

Kondrashina et al. [124] reported that GLP-1 formation increased after 6 months of ripening of Irish cheddar cheese, but the stimulating effect of the GLP-1 was lost during the digestion and a protective factor was needed. Gillespie and Green [125] reported that caseins do not exert cytotoxic effects on enteroendocrine cells and that α- and β-casein, in particular, enhance GLP-1 secretion; however, this effect is diminished following proteolytic hydrolysis. Given that proteolysis is a key process during cheese ripening, these findings suggest that GLP-1 responses may be modulated throughout the ripening period. In another study investigating the water-soluble peptide fractions of Gouda-type cheese, DPP-4 inhibitory activity was shown to increase during the ripening process. The β-casein-derived peptide LPQNIPPL was identified as the primary contributor to this activity, and was suggested to exhibit a potential effect on the incretin system by improving glucose tolerance in vivo through a mechanism involving the limitation of GLP-1 degradation [126]. In another study by Wei et al. [127], the effect of production technique on bioactive peptides of Rubing, a traditional Chinese cheese, produced by traditional and fermentation acidification techniques, was investigated. In vitro assay results showed that the cheeses by fermentation acidification technique had higher ACE-I, α-glucosidase inhibitor and antioxidant activity than those by traditional technique during the digestion.

On the other hand, bioactive peptides can also be found in whey, a milk product [128]. Corrêa et al. [128] determined the antioxidant and ACE inhibitory activities of sheep cheese whey by hydrolysis with proteases and hydrolysates from *Bacillus* sp. P7. It was stated that the ACE inhibitory activities of sheep cheese whey by the hydrolysis reached maximum level, and the application can significantly contribute to the dairy industry. Similarly, in another study, bioactivity and peptide profiles of whey protein hydrolysates from Colombian cheese were investigated, and the results showed that higher antioxidant and ACE inhibitory capacities and higher bioavailability of peptides after in vitro gastrointestinal digestion were detected [129].

In another study, the effect of industrial and natural starter cultures on bioactive peptides in Asiago-PDO cheese during ripening was investigated [130]. The researchers found that density of bioactive peptides differed during ripening due to proteolysis and that peptide profile changes. The natural microbiota was found to produce more dominant ACE inhibitors, and the importance of the region for traditional cheese production was emphasised. Öztürk and Akın [118], in a study on peptide profile of Tulum cheese produced traditionally in the Taurus region of Turkey, stated that peptide numbers increased at end of 180 days and that this increase may be due to microbial proteolytic activity (intracellular and extracellular enzymes) during ripening. Similarly, Pisanu et al. [131] stated that cheeses produced with raw milk had higher ACE inhibitor peptides than those with pasteurised milk.

### Fatty Acids

Fatty acids play an important role in the flavour of cheese by playing a leading role in catabolic reactions that release flavour components such as lactones, esters, methyl ketones, and secondary alcohols [132]. Therefore, their presence in cheese is of great importance as a postbiotic during the ripening. Balthazar et al. [95] investigated the effects of flavour enhancers (arginine, yeast extract, and oregano extract) and probiotics (*L. casei*) on the metabolite profile of low-sodium Prato cheese. The results showed that sodium reduction did not significantly affect the metabolic profile of the cheeses, whereas the addition of yeast extract specifically promoted lipolysis, leading to an increase in fatty acid content. These fatty acids, including short- and medium-chain fatty acids, are important as they act as precursors of flavour compounds such as lactones, esters, methyl ketones, and secondary alcohols, thereby contributing to cheese flavour development.

The fermentation of non-digestible carbohydrates into short-chain fatty acids (SCFA), primarily acetate, propionate, and butyrate, is another significant function of probiotics. Numerous studies have demonstrated the beneficial effects of these substances on human energy metabolism, health and defence against inflammatory bowel conditions, obesity, colorectal cancer, and other illnesses [133, 134]. Ruiz-Moyano et al. [135] evaluated the probiotic potential of LAB strains isolated from Serpa cheese and reported that certain strains, including *L. brevis* C1Lb21, were capable of producing SCFAs such as acetic, propionic, and butyric acids during fermentation of prebiotic substrates. These metabolites are important, as SCFAs are known to play a key role in gut health, energy metabolism, and protection against inflammatory and metabolic disorders.

In another study, Cheddar cheese produced with probiotic strains including *B. longum* 1941, *B. lactis* LAFTIs B94, *L. casei* 279, *L. paracasei* LAFTIs L26, and *L. acidophilus* strains showed enhanced metabolic activity during ripening. Specifically, certain strains (e.g., *L. casei* 279 and *L. paracasei* LAFTIs L26) exhibited increased proteolytic activity, resulting in greater casein hydrolysis and higher levels of free amino acids, while others contributed to increased production of organic acids such as acetic acid. These changes are important as they contribute to the development of cheese flavour and overall quality [136].

### γ-aminobutyric Acid

GABA, the most significant inhibitory neurotransmitter in the central nervous system, has many functional properties such as improving brain function, lowering blood pressure, reducing stress, inducing insulin secretion, and protecting β-cells [137]. In another study by Li et al. [138], the effect of yeast addition to Kazakh cheese, a traditional Chinese dairy product, was investigated, and it was found that the addition of *Kluyveromyces marxianus* B13-5 increased the GABA content in the cheese (95.6 mg/100 g). GABA has been reported in various traditional cheeses, with its concentration strongly depending on microbial activity during ripening. For instance, in mould-ripened Civil cheese, GABA levels exceeding 1000 mg/kg were detected in the majority of samples (17 out of 25), highlighting cheese as a significant dietary source of this bioactive compound. The production of GABA in cheese is mainly associated with LAB possessing glutamate decarboxylase (GAD) activity, such as *L. plantarum*, which can produce high amounts of GABA (up to 626 mg/L under laboratory conditions). These findings indicate that the complex microbiota of traditional cheeses plays a crucial role in converting glutamate into GABA during ripening [139, 140].

### Antimicrobial Metabolites

Natural antimicrobial metabolites, classified as postbiotics, have numerous advantages for food safety and human health. In a study on the extension of the shelf life of spreadable Ricotta cheese by the addition of inulin and *L. paracasei* BGP1, it was found that the production of sufficient amounts of antimicrobial compounds contributed to the extension of the shelf life of the product [108]. In another study by Langa et al. [110], different combinations of *L. lactis* INIA 650, *L. paracasei* INIA P272 and *B. breve* INIA P734 were used as co-cultures for cheese production. It was determined that nisin, an antimicrobial peptide, was produced by *L. lactis* INIA 650 both during cheese production and ripening. Furthermore, it was found that the nisin was also produced in an in vitro colon model and that using multi-strain combinations in cheese production could improve both cheese quality and intestinal health.

## Kefir as a Potential Postbiotic Source

Kefir, an important fermented milk product consumed worldwide, is a potential source of probiotic microorganisms since starter culture or kefir grains are used in its production [141, 142].

### Exopolysaccharides

Microbial EPS affect the sensory properties of fermented products due to their functional characteristics, such as emulsification, viscosity, and thickening [143]. Kefir exopolysaccharides, known as ‘kefiran’, can be isolated from kefir granules [144]. The EPS amount, water-holding capacity, and hardness of kefir samples produced with cow and buffalo milk were investigated by Gül et al. [145]. The results showed that the EPS amount by starter culture in kefirs produced with buffalo milk was higher than that produced with cow milk. The higher EPS production observed in kefir made from buffalo milk may be attributed to its richer composition, particularly its higher protein and total solids content, which can enhance microbial metabolism and EPS synthesis. In addition, differences in the native microbiota of buffalo milk may influence the activity of EPS-producing microorganisms, thereby contributing to the observed variation. Furthermore, the researchers stated that there was a positive correlation between EPS amount and water-holding capacity.

### Organic Acids and Volatile Compounds

Organic acids produced during fermentation and storage directly affect the sensory characteristics of fermented products and consumer acceptance, and they are critical for product flavour [146]. For example, in kefir, the metabolic activity of LAB and yeasts leads to the formation of various organic acids, including lactic, pyruvic, acetic, and citric acids, as well as methanol [146, 147]. Due to their significant contribution to flavour and overall product quality, numerous studies have focused on isolating and characterising kefir-associated microorganisms to standardise production, enhance volatile compound formation, and improve organoleptic properties [148, 149].

### Antimicrobial Metabolites

**Fig. 4:**
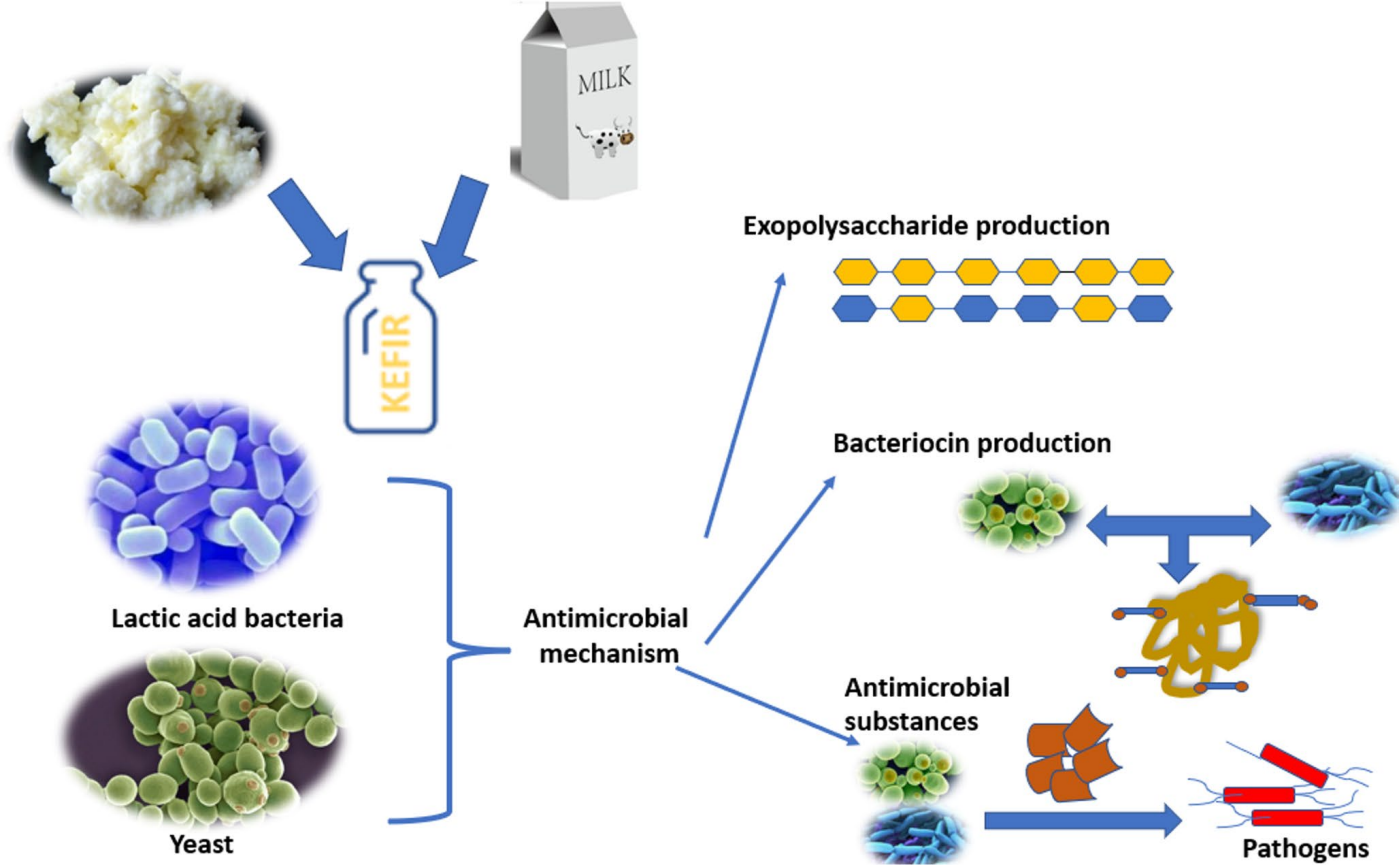
Antimicrobial metabolites produced in kefir. Schematic representation of kefir-associated microorganisms, including LAB and yeasts, and their role in producing antimicrobial metabolites such as organic acids, bacteriocins, and exopolysaccharides, which inhibit the growth of pathogenic microorganisms

Kefir contains various antimicrobial metabolites such as organic acids, peptides, and bacteriocins, and the interaction between these compounds may enhance their overall antimicrobial activity against foodborne pathogens [150]. The antimicrobial effects of these metabolites are illustrated in Fig. 4. Compounds such as lactic acid, hydrogen peroxide, and bacteriocins produced by kefir-associated microorganisms are known to inhibit the growth of foodborne pathogens, including *Bacillus cereus*, *Clostridium tyrobutyricum*, *E. coli*, *L. monocytogenes*, and *S. aureus*, particularly within fermented food matrices and in vitro conditions [151, 152]. Santos et al. [153] stated that lacticin 3147 by *L. lactis* DPC3147 isolated from kefir grains had antimicrobial activity on *E. coli*,* L. monocytogenes*, *S.* Typhimurium, and *Salmonella* Enteritidis. In addition, Miao et al. [154] reported that bacteriocin F1 by *L. paracasei* ssp. *tolerance* from Tibetan kefir has a wide range of antimicrobial spectrum against *E. coli*,* Salmonella enterica*,* Shigella dysenteria*,* S. aureus*,* Bacillus thuringiensis*,* Aspergillus niger*, and *Rhizopus nigricans*.

Recently, some researchers found that *L. paracasei* FX-6 could produce antimicrobial agents that affect the activity of pathogenic *Pseudomonas putida* [155]. On the other hand, in the study by Ghane et al. [156], cell-free supernatant from *L. paracasei* LAB2 and LAB4 inhibited biofilm formation and growth of *E. coli*. However, the chemical structures of these antimicrobial metabolites, particularly bacteriocins and other bioactive compounds produced by kefir-associated microorganisms, have not yet been fully determined. Therefore, further studies are needed to elucidate the nature of antimicrobial components. Studies on the inhibitory activity of isolates from kefir against foodborne pathogens are listed in Table 3.

**Table 3:** Inhibitory activity of antimicrobial metabolites produced by kefir strains against foodborne pathogens

Kefir strains	Pathogens	References
*L. kefiri* CIDCA 8348	*Pseudomonas aeruginosa*, *Salmonella* Enteritidis, *Shigella flexneri*	[157]
*L. plantarum* 299 V	*E. coli*,* Salmonella* Typhi, *S. aureus*	[158]
*Kazachstania unispora* M3	*E. coli*,* P. aeruginosa*,* Salmonella* sp.	[159]
*Lactobacillus* spp.	*S.* Typhimurium	[155]
*L. lactis*, *Kazachstania unispora*, *S. cerevisiae*	*E. coli*,* S.* Typhimurium, *S. aureus*	[160]
*Lactobacillus diolivorans* 1Z	*S.* Typhimurium	[161]
*L. kefiri*	*S.* Enteritidis	[162]
*L. kefiranofaciens* DN1	*L. monocytogenes*,* S.* Enteritidis	[163]

## Fermented Milk as a Potential Postbiotic Source

Fermented milk is the product formed as a result of the acidification of milk through LAB activity, which causes significant physicochemical, sensory, and microbiological changes in fermented dairy products. In recent years, probiotic bacteria have been added to fermented milk to satisfy consumer demand. The health-promoting properties of milk and dairy products are essentially associated with postbiotics. During milk fermentation by LAB, various bioactive compounds are produced, including free amino acids (FAAs), free fatty acids, organic acids, vitamins, bioactive peptides, and oligosaccharides [164]. In addition to increasing the content of biofunctional components, the fermentation also changes the colloidal and structural properties of the milk. LAB hydrolyse milk proteins to produce peptides and use lactose as a carbon source to produce organic acids (especially lactic acid), both of which affect the colloidal structure of milk [165].

### Organic Acids in Fermented Milk

Milk and dairy products contain minerals, vitamins, enzymes, FAAs, and organic acids. Lactic, citric, orotic, sialic, benzoic, and sorbic acids are the main organic acids in milk [166]. During fermentation, LAB produce lactic acid from lactose, increasing the acidity of products and enabling the breakdown of milk proteins [167]. Lactic and acetic acids are decomposition products of lactose, and lactic acid has been shown to exhibit antimicrobial activity. For example, Wang et al. [168] reported that 0.5% lactic acid inhibited the growth of pathogens such as *Salmonella* spp., *E. coli*, and *L. monocytogenes*. Similarly, Akbar et al. [169] demonstrated that lactic acid produced by LAB in fermented milk exhibited antimicrobial activity against *S*. Typhimurium, *S. aureus*, *E*. *coli*, and *L. monocytogenes*. In addition, benzoic and sorbic acids are known to inhibit the growth of various microorganisms in milk [170] and contribute to improving product quality during storage.

### Bioactive Peptides

**Fig. 5:**
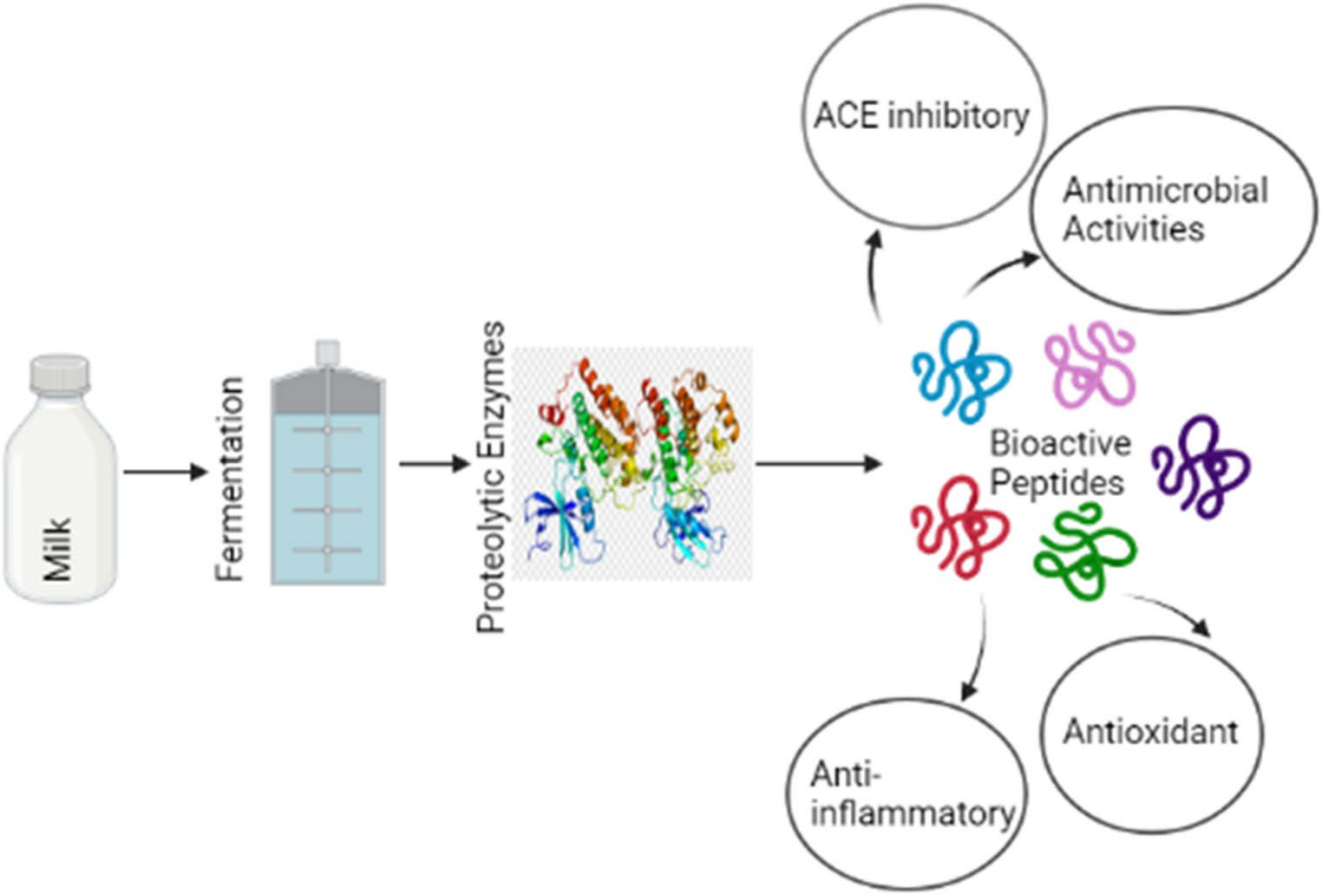
Production of bioactive peptides in fermented milk. Overview of the formation of bioactive peptides during milk fermentation via proteolytic activity of microorganisms, and their subsequent biological functions, such as ACE inhibition, antimicrobial activity, antioxidant effects, and anti-inflammatory responses

Peptides with different bioactivities are present in fermented milk [171] and can be produced by digestion in the gut or by enzymatic proteolysis during food processing [172]. Figure 5 shows the production of bioactive peptides in fermented milk.

The health benefits of bioactive peptides have been associated with their numerous biological activities, including antioxidant, antihypertensive, antithrombotic, antimicrobial, immunomodulatory, and antitumour [173]. Examples of bioactivity in fermented milk are summarised in Table 4. Several anti-hypertensive peptides produced in milk fermentation have potent activity against ACE [174].

**Table 4:** Bioactive peptides in fermented milk and their bioactivity

Microorganisms	Bioactive peptides	Bioactivity	References
*L*. *delbrueckii* subsp. *bulgaricus ORT2*,* L. reuteri SRM2*,* L. lactis* spp. *lactis* BRM3	Peptides fraction	ACE inhibitor, antioxidant activity	[175]
*L. lactis* NRRL B-50571	Peptides fraction	ACE inhibitor	[176]
*L. delbrueckii* QS306	Peptides fraction	ACE inhibitor	[177]
*Bifidobacterium infantis*,* L. acidophilus* ATCC 4356, *L. brevis* ATCC 14869, *L. casei*,* Latilactobacillus curvatus* ATCC 25601, *L. helveticus* ATCC 15009, *L. helveticus* CNRZ32, *L. helveticus* 89, *Lactobacillus jensenii* ATCC 25258, *L. rhamnosus* ATCC 7469, *L. reuteri* ATCC 55730	Casein hydrolysates	ACE inhibitory	[178]
*L. plantarum*	Peptide fraction	Anti-inflammatory activity, antihemolytic activity, antioxidant activity, antimutagenic activity, antimicrobial activity	[179]
*L*. *delbrueckii* subsp. *bulgaricus*,* E. faecalis TH563*	Peptide fraction	ACE inhibitor	[180]
*L. lactis DIBCA2*	Peptide fraction	ACE inhibitor	[181]
*E. faecalis*	Peptide fraction	ACE inhibitor	[182]

In a study by Loghman et al. [175], single and co-cultures of proteolytic *L*. *delbrueckii* subsp. *bulgaricus ORT2*,* Limosylactobacillus reuteri* SRM2, and *L. lactis* spp. *lactis BRM3* isolated from different sources were used for fermented milk production. It was determined that peptide fractions by the bacteria had high ACE inhibitory and antioxidant activity. Nevertheless, the magnitude of these effects may vary depending on strain combinations and fermentation parameters, and standardised comparisons across studies are currently lacking. On the other hand, Álvarez-Olguín et al. [176] evaluated the bioaccessibility and bioavailability of ACE-inhibitory peptides derived from fermented milk containing *L. lactis* NRRL B-50571, highlighting that the physiological effectiveness of these peptides depends not only on their in vitro activity but also on their stability during digestion and absorption in the gastrointestinal tract. The results showed that the peptides retained their ACE inhibitory activity after digestion.

## Ice Cream as a Potential Postbiotic Source

Ice cream, defined by many researchers as a frozen dessert or a dairy product, is a product that is loved and consumed by everyone worldwide [183]. Various supplements, including plant extracts, pulp, or probiotics are added to ice cream to enhance its functional properties [184, 185]. Moreover, ice cream, like other dairy products, serves as a carrier for probiotic bacteria [102].

Postbiotics have recently gained increasing attention as an alternative or complement to probiotic microorganisms [179, 186, 187]. Postbiotic usage in foods, including ice cream, is very popular due to its beneficial effects [188]. Although there are many studies on probiotic usage in the ice cream, studies on postbiotics (EPS only) are limited [186, 187]. Probiotic microorganisms in ice cream are listed in Table 5.

**Table 5:** Overview of probiotic microorganisms used in ice cream formulations

Microorganisms	References
*L. rhamnosus*	[62–66, 106, 107, 109, 157–163, 177, 178, 180–182, 185, 189–198]
*L. casei*	[182, 189–201]
*E. faecium*	[202]
*L. paracasei*	[198]
*Limosilactobacillus fermentum*	[203, 204]
*B. animalis* spp. *lactis* BB-12	[205, 206]
*L. acidophilus*	[195, 198], 207, 208, 209, 210, 211]
*S. boulardii*	[193, 212–214]
*L. plantarum*	[215–217]
*B. breve*	[217, 218]
*B. animalis*	[194, 218, 219]
*Bifidobacterium lactis*	[220, 221]
*B. bifidum*	[200, 222]
*L. brevis*	[223]
*B. longum*	[200, 224]
*L. plantarum*	[225]
*L. paracasei*	[196, 200]
*Lactobacillus* sp.	[226]
*Bifidobacterium adolescentis*	[201]

### Exopolysaccharides

**Fig. 6:**
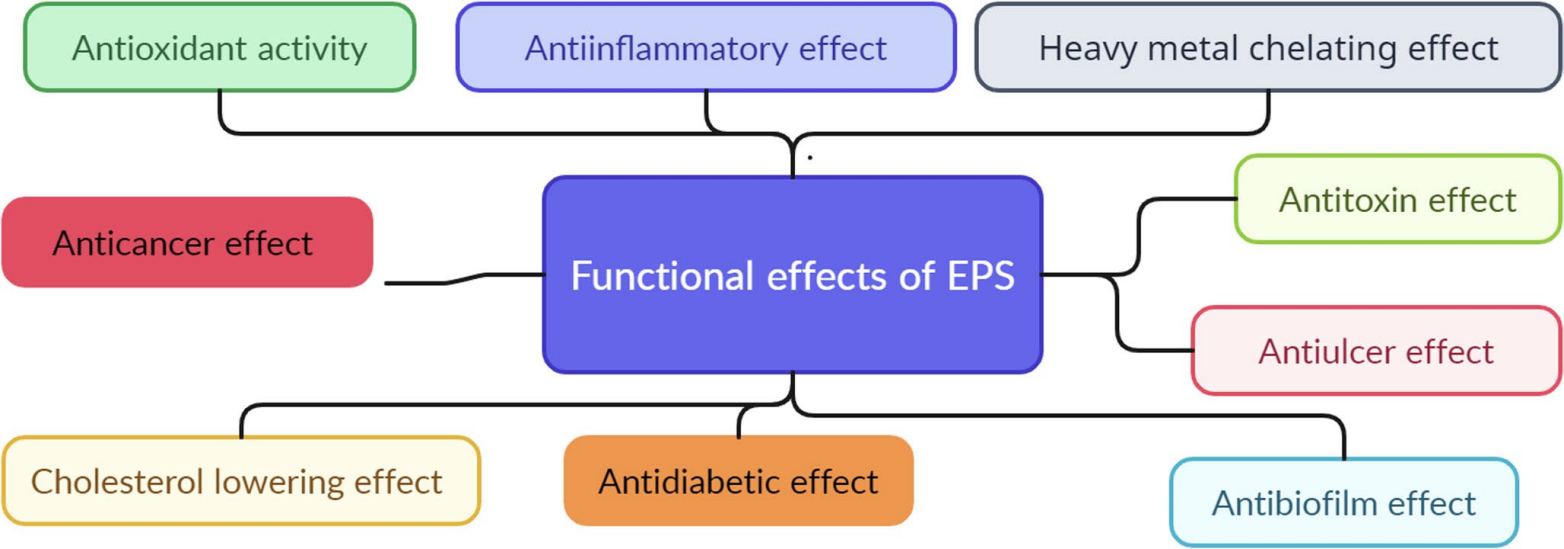
Beneficial effects of EPS on human health. Overview of the functional roles of microbially derived EPS, highlighting their wide range of biological activities such as antioxidant, anti-inflammatory, anticancer, antidiabetic, cholesterol-lowering, antibiofilm, antiulcer, antitoxin, and metal-chelating effects [187]

Exopolysaccharides (EPS) play an important role in the physicochemical, textural, rheological, and sensory properties of ice cream, while also exhibiting various functional properties beneficial to human health, including antioxidant, antimicrobial, and anticancer activities (Fig. 6) [227]. Usage of EPS is possible as a stabiliser, emulsifier, gelling, and viscosifying agent, to enrich nutrition, texture, rheology, water-holding capacity, and palatability in ice cream [25, 227, 228]. In the study by Dertli et al. [186], it was investigated the effects of EPS by probiotics on the rheological, physicochemical, and sensory properties of ice creams and results showed that EPS-containing ice creams had better rheological properties (consistency coefficient and apparent viscosity). Moreover, it was stated that EPS-containing ice creams can be produced without adding stabiliser and the ice creams can have high quality characteristics. Goh et al. [229] investigated the effects of metabolites by EPS-producing *L. delbrueckii* subsp. *bulgaricus* LB18 and *L. delbrueckii* subsp. *bulgaricus* CNRZ Z737 on some properties of ice cream. They determined that the strains improved characteristics of ice cream such as overrun, resistance to meltdown, heat shock tolerance, and scoopability. Moreover, it was determined that these strains could be used instead of stabiliser substances. In other study, the effects of EPS by *L. plantarum* YW11 on some quality properties (viscosity, firmness, overrun, destabilised fat, and melting behaviour) of ice cream were investigated. It was stated that the EPS could be used to improve viscosity, firmness, overrun, destabilised fat, and melting properties of ice cream [187]. Fragoso et al. [230] used thermotolerant probiotic *P. pentosaceus* UAM22 in fat reduced ice cream. It was determined that the ice creams with *P. pentosaceus* UAM22 had a more adhesive and softer texture along with improved melting properties. Consequently, studies on the use of postbiotics in ice cream, such as short-chain fatty acids, GABA, and organic acids, are still limited. Further research is needed to explore the potential applications of postbiotics in ice cream production.

## Conclusion

The potential importance of postbiotics in the dairy industry is becoming increasingly evident. As demonstrated in this review, postbiotics derived from LAB and other fermentative microorganisms offer a unique combination of health benefits and technological advantages, making them ideal candidates for functional dairy products. The stability, safety, and diverse bioactive properties of these substances position them as promising alternatives to conventional functional ingredients or complements to probiotic microorganisms in dairy products. However, further research is needed to standardise production processes, clarify dose-response relationships, and validate health claims through rigorous clinical trials. It is recommended that future studies explore the synergistic effects of postbiotics with other bioactive compounds and their applications in the field of personalised nutrition. Despite promising findings, the translation of postbiotic effects into clinically validated health outcomes remains limited and requires further investigation. It is submitted that by addressing the challenges related to the standardisation of production processes, clarification of dose-response relationships, and validation of health claims through rigorous clinical trials, the dairy industry has the potential to harness the full potential of postbiotics. This approach, in turn, could result in the creation of innovative products that promote consumer health. Furthermore, this advancement is expected to meet the demands for quality, safety, and sustainability.

## Data Availability

No datasets were generated or analysed during the current study.
